# Phenotypic Consequences *In vivo* and *In vitro* of Rearranging the P Gene of RABV HEP-Flury

**DOI:** 10.3389/fmicb.2017.00120

**Published:** 2017-02-03

**Authors:** Mingzhu Mei, Teng Long, Qiong Zhang, Jing Zhao, Qin Tian, Jiaojiao Peng, Jun Luo, Yifei Wang, Yingyi Lin, Xiaofeng Guo

**Affiliations:** ^1^College of Veterinary Medicine, South China Agricultural UniversityGuangzhou, China; ^2^Key Laboratory of Zoonosis Prevention and Control of Guangdong ProvinceGuangzhou, China

**Keywords:** Rabies virus, HEP-Flury, gene rearrangement, phosphoprotein, pathogenicity

## Abstract

Phosphoprotein (P) of the Rabies virus (RABV) is critically required for viral replication and pathogenicity. Here we manipulated infectious cDNA clones of the RABV HEP-Flury to translocate the P gene from its wild-type position 2 to 1, 3, or 4 in gene order, using an approach which left the viral nucleotide sequence unaltered. The recovered viruses were evaluated for the levels of gene expression, growth kinetics in cell culture, lethality in suckling mice and protection of mice. The results showed that viral replication was affected by the absolute value of N protein which was regulated by P protein. Viral lethality in suckling mice was consistent with the ratio of P mRNA in one complete transcription. The protection of mice induced by viruses was related to the antibody titer 5 weeks post-infection which might be regulated by G protein. However, the ability to induce cell apoptosis and viral spread were not only related to the viral replication but also to the ratio of related gene which affected by the gene position. These findings might not only improve the understanding of phenotype of RABV and P gene rearrangement, but also help rabies vaccine candidate construction.

## Introduction

Rabies is a zoonotic disease caused by rabies virus (RABV) belonging to the *Lyssavirus* genus of the *Rhabdoviridae* family, *Mononegavirales* (Pringle, 1996). It is still a major health concern in many developing countries. The RABV genome is approximately 12 kb encoding five proteins: nucleoprotein (N), phosphoprotein (P), matrix protein (M), glycoprotein (G), and the RNA-dependent RNA polymerase (L) (Albertini et al., 2011). RABV P is a multifunctional protein: it is an essential cofactor of the virus RNA-dependent RNA polymerase which is important for the genome transcription/replication, and in addition, it has been identified as an interferon antagonist (Blondel et al., 2002; Brzozka et al., 2005; Vidy et al., 2005; Rieder et al., 2011). The reduced expression of P protein can decrease the ability to prevent IFN induction (Brzozka et al., 2005; Marschalek et al., 2009) and a def-P virus has been demonstrated apathogenic in both adult and suckling mice, even when inoculated intracranial (Shoji et al., 2004; Morimoto et al., 2005). Moreover, wild-type RABV P protein has been reported to assist viral replication in muscle cells by counteracting the host IFN system, consequently, enhancing infection of peripheral nerves (Niu et al., 2013; Yamaoka et al., 2013). Also, P protein can interact with mitochondrial complex I and induces mitochondrial dysfunction and oxidative stress (Kammouni et al., 2015).

Gene rearrangement can alter the genotype of a virus, resulting in a predictable change in gene expression which would be invaluable for studies of gene be invaluable for studies of gene function and control (Wertz et al., 1998). Previous studies have successfully rearranged the five viral genes of vesicular stomatitis virus (VSV), a prototype *Vesiculovirus* genus of the *Rhabdoviridae* family, and recovered viable viruses from each of the rearranged cDNAs (Wertz et al., 1998; Ball et al., 1999). Subsequently, they demonstrate that neither of the RNA species in variant viruses infecting cells nor the relative molar ratios of the proteins in mature virus particles are changed by gene rearrangement. Gene rearrangement only affects the relative levels of protein expression and consequently alters the phenotypes and lethality in mice infected with recombinant viruses (Wertz et al., 1998; Ball et al., 1999; Flanagan et al., 2000).

Unlike VSV, which is a highly cytopathic virus and should replicate very fast, RABV regulates viral gene expression to produce viral components in sufficient amounts for viral spread, but low enough to maintain host cell survival and to escape from antiviral host cell responses (Albertini et al., 2011). In previous work, G mRNA of RABV ERA can be increased 30% by switching the positions of G gene with M gene (Wu and Rupprecht, 2008). However, the high-egg-passage Flury (HEP-Flury) strain, one of the most attenuated of rabies fixed strains used as a vaccine for humans in Japan, could produce more translatable P and G, less M, and equivalent L mRNA compared to ERA which transcription mode like VSV (Morimoto et al., 1992; Morimoto et al., 2011). And RABV P is important for the viral pathogenicity and antiviral response (Chopy et al., 2011; Niu et al., 2011, 2013; Rieder and Conzelmann, 2011; Fouquet et al., 2015). Therefore, investigation of P gene rearrangement can contribute to development of rabies vaccine.

In previous work, we have rearranged the M gene of RABV HEP-Flury from its wild-type position 3 to 2 or 4 decreasing its replication in BSR cells (Yang et al., 2014). Here, we first rearranged the P gene order of HEP-Flury, translocating it from wild-type position 2 to 1, 3 or 4 and subsequently recovered the infectious viruses. We did this investigating the relationship between gene transcription/expression and viral phenotype caused by P gene rearrangement thus provided an optional approach to rabies vaccine development. The results showed that the viral lethality for suckling mice was in accord with the ratio of P mRNA in one complete transcription which decreased as its gene was moved successively away from promoter proximal position to successive positions down the viral genome. Importantly, these changes occurred leaving the protection of mice intact or better, suggesting that this approach may provide a rational method to achieve a measured and stable degree of attenuation of this type of virus. Moreover, since the *Mononegavirales* have not been observed to undergo homologous recombination, gene rearrangement should be irreversible (Pringle, 1982).

## Materials and Methods

### Mice

Specific pathogen-free (SPF) female Kunming adult and pregnant mice were purchased from Center for Laboratory Animal Science, the Southern Medical University (Guangzhou, China). They were housed under specific-pathogen-free conditions in biosafety level containment in the Laboratory Animal Center of South China Agricultural University. All procedures involving animals and their care were conducted in conformity with NIH guidelines (Care and Animals, 2011) and approved by the Animal Care and Use Committee of the South China Agricultural University.

### Viruses and Cells

Recombinant RABV rHEP-Flury and P3 with the P gene in position 3 was previously rescued in our laboratory via reverse genetics (Yang et al., 2014). Baby hamster kidney cells (BHK-21) were used for virus recovery from cDNA and cultured in Dulbecco modified essential medium (DMEM) (Gibco, China), supplemented with 10% fetal bovine serum (FBS) (Gibco, Australia). Mouse neuroblastoma NA cells which were used to amplify recombinant viruses and subsequent experiments, were grown in RPMI 1640 medium (Gibco, China) supplemented with 10% FBS.

### Plasmid Construction and Recovery of Recombinant Viruses

The plasmid pHEP-3.0 containing the full-length genomic cDNA of HEP-Flury and four helper plasmids pH-N, pH-P, pH-G, and pH-L were a kind gift from Dr. Kinjiro Morimoto. For detailed information about the plasmids, please refer to Inoue et al. (2003).

Construction of a full-length cDNA clone of the P gene rearranged genome and recovery of infectious viruses has been described previously (Ghanem et al., 2012; Wang et al., 2014; Yang et al., 2014; Luo et al., 2016). All the genes were rearranged from the beginning of the transcription start site (AACA) to the transcription end signal (the poly A signal, AAAAAAA). To rearrange the P gene of RABV without introducing any additional changes into the viral genome, we used inverse PCR to amplify the linearized vector for the P1 plasmid with the P gene in position 1 via primers 1–5′VR (5′-*ACATTTTTGCTTTGCAACTGACGATGTC*-3′; the 15–20 bp homologous sequences are underlined) and 1–3′ VF (5’–*AGGCAACACCACTAATAAAATGAAC*–3′; the 15-20 bp homologous sequences are underlined). The linearized vector for the P4 plasmid with the P gene in position 4 was synthesized using primers 4–5′VR (5′-*AGTTTTTTTCATGATGGATATACACAATC*-3′; the 15–20 bp homologous sequences are underlined) and 4–3′VF (5′-*TGTATACCAAAAGAACAACTAACAACAC*-3′; the 15–20 bp homologous sequences are underlined). The primers for the amplification of genes are list in **Table 1**. To avoid mutation, a Phusion High-Fidelity DNA Polymerase (Thermo Scientific, USA) was used following the manufacturer’s instructions. Then an efficient homologous-recombinant-based ClonExpress^TM^ MultiS one step cloning method was adopted according to manufacturer’s instructions (Vazyme Biotech, Nanjing, China). After plasmid sequencing, the plasmids of the rearranged cDNAs and the four helper plasmids pH-N, pH-P, pH-G, and pH-L were used to co-transfect BHK21 cells via the SuperFect Transfection Reagent (Qiagen, USA) according to manufacturer’s instructions. Twelve days later, we collected the supernatants of transfected cells and examined the existence of the rescued virus via direct immunofluorescence assay (IFA). Subsequently, the viruses rescued successfully were passaged in NA cells.

**Table 1:** Oligonucleotides used for construction of RABV genome cDNAs^a^.

Primer name	Sequence (5′–3′)	Use
1-P-F	GCAAAGCAAAAATGTAACACTCCTCCTTTCGAACC	Construction for P1 plasmid
1-P-R	ATTGTAGGGGTGTTAGTTTTTTTCATATCGACTCC	
1-N-F	TAACACCCCTACAATGGATGCCGAC	
1-N-R	TTAGTGGTGTTGCCTGTTTTTTTCATGATGGATATAC	
4-M-F	ATCATGAAAAAAACTAACACCACTAATAAAATGAAC	Construction for P4 plasmid
4-M-R	TGAGGGATGTTGCCTGTTTTTTTCACATCCAAGAGGC	
4-G-F	AGGCAACATCCCTCAAAAGACTTAAGG	
4-G-R	GGAGGAGTGTTAATAGTTTTTTTCTCGACTGAAATG	
4-P-F	TATTAACACTCCTCCTTTCGAACCATCC	
4-P-R	GTTCTTTTGGTATACAGTTTTTTTCATATCGACTCCATG	

*^a^15–20 bp homologous sequences are underlined.*

Following eight passages in NA cell culture, the gene order of the recovered viruses was determined via reverse transcription (RT)-PCR using three pairs of primers: DF (5′-*CTTAACAACAAAACCAAAGAAGAAGCA*-3′) and PR (5′-*CATCTCAAGATCGGCCAGACCG*-3′); DF and NR (5′-*TGAAGTTCGGTATAGTACTCC*-3′); and DF and MR (5′-*GTCCTCATCCCTACAGTTTTTC*-3′). Subsequently, the PCR fragments were sequenced directly.

### Lethality in Suckling Mice

The lethality of individual virus was measured in suckling Kunming mice aged 1-day to 3-days, obtained from the Southern Medical University of China. Groups of twelve mice were intracranial (IC) inoculated either with 20ul diluent RPMI 1640 or with serial 10-fold dilutions of individual virus, and then observed daily. The titers of viruses were diluted to 10^5.5^FFU/ml before serial dilutions and any mouse dying within 4 days post-inoculation was ignored. The LD_50_ for each virus was calculated via the method of Reed and Muench.

### Protection of Mice

Groups of 10 mice aged 6-weeks to 8-weeks were immunized once intramuscularly (IM) with different doses of either rHEP-Flury or one of the variant RABVs. To determine antibody levels, blood samples were collected 21 days post immunization. Serum samples were pooled and heated to 57°C for 30 min to inactivate complement. Mice were then challenged IC with 50 LD_50_ of Challenge Virus Standard (CVS-24) and observed daily for a 28 day period. Survivor numbers were recorded and any mouse dying within 4 days post-challenge was ignored.

### Monitoring Antibody Levels in Mice

Groups of five mice aged 6-weeks to 8-weeks old were inoculated IM with 10^5^ FFU individual viruses. Subsequent to virus inoculation, blood was collected at weekly intervals. Serum samples were pooled and heated to 57°C for 30 min to inactivate complement. The serum antibody titers were monitored using a Serelisa^®^ Rabies Ab Mono Indirect kit (Synbiotics, France) following manufacturer’s introductions. For calculated titer >0.6EU/ml, the animal is considered as protected.

### One-Step and Multi-Step Growth Analyses of Viruses in NA Cells

NA cell monolayers were infected with rHep-Flury and variant viruses at a multiplicity of infection (MOI) of 0.01 for multi-step growth curves and a MOI of 3 for one-step growth curves. After 1h of adsorption at 37°C, the inoculum was removed, cells were washed with phosphate buffered saline (PBS) Thermo scientific, China) twice, and 5 ml of fresh PRMI medium containing 5% FBS was added and incubated at 34°C. Samples were harvested at indicated intervals over a 120 h period, and viral titers were quantified via direct fluorescent antibody test (FAT) as described previously described on NA monolayers (Zhao et al., 2009).

### Viral Spread in NA Cells

NA cell monolayers were infected with rHep-Flury and variant viruses at a MOI of 0.005, then incubated for a 72h period at 34°C, and stained every 12 h with FITC Anti-Rabies Monoclonal Globulin (Fujirebio, Malvern, PA, USA), before they were examined under a fluorescence microscope(Wirblich and Schnell, 2011).

### Cell Apoptosis by Flow Cytometry

Cell apoptosis was quantified by using an Annexin V-FITC apoptosis kit (BestBio, China) according to the manufacturer’s instructions. NA cells were seeded into 6-well plates and incubated at 37°C overnight. Then cells were treated with RABV rHEP-Flury and P gene rearranged viruses at a MOI of 3. Twenty-four hours later, cells were collected and incubated with 5 μl Annexin V-FITC and 10 μl PI for 15 min. Finally, 500 μl of binding buffer was added to each tube and analyzed by a Beckman FC 500 flow cytometry(Beckman Coulter, Fullerton, CA, USA), followed by data analysis with the corresponding CXP Software.

### RNA Isolation and qRT-PCR

A MOI of 3 was chosen to make sure every cell was infected. Monolayer of NA cells grown in six-well plates were infected with rHep-Flury and variant viruses, respectively, and incubated at 34° C for 12 h. Cells were then washed once with PBS, and RNA was isolated using HiPure Universal RNA Kits (Magen, Guangzhou, China) following the manufacturer’s instructions at the indicated intervals. For the viral structural gene expression, cDNAs were synthesized with oligo/ (dT_23_) primer using the HiScript^®^ II 1st Strand cDNA Synthesis Kit (Vazyme Biotech, Nanjing, China). For the quantification of leader RNA, cDNA was synthesized with a tagged primer with attached 18-nucleotide (nt) tag that was unrelated to RABV as previous described (Yang et al., 2015a). For the quantification of genomic RNA (vRNA), cDNA was synthesized with N-QF. **Table 2** provides primer sequence details. The real-time SYBR Green PCR assay was carried out in a CFX384 Real-time System (Bio-Rad, USA) using Universal SYBR Green Master (Vazyme Biotech, Nanjing, China) according to the manufacturer’s instructions. Numbers of RNAs copies of a particular gene were normalized in relation to the housekeeping gene beta actin (β-actin).

**Table 2:** Oligonucleotides used for quantification of RABV structural gene and leader RNA.

Primer name	Sequence (5′–3′)	Use
N-QF	TAGGCTTGAGTGGGAAGTC	qRT-PCR/cDNA synthesis
N-QR	CAGCAATAACCGTGGCAT	qRT-PCR
P-QF	ATCGCTCATCAGATTGCT	qRT-PCR
P-QR	GCCTCTTTAACTATGTCATCAA	qRT-PCR
M-QF	GAACATACGGGCTTAACTCC	qRT-PCR
M-QR	AAGAGGCTCAAAATGTAACGG	qRT-PCR
G-QF	TCTACAGTTTTCAAAGACGG	qRT-PCR
G-QR	TCAATACATACTTCCCCCAT	qRT-PCR
L-QF	TGTTGATGTCTGATTTCGC	qRT-PCR
L-QR	GGAACGCTCTTGACAGAT	qRT-PCR
β-ACTIN-QF	CGTAAAGACCTCTATGCCAAC	qRT-PCR
β-ACTIN-QR	GATCTTGATCTTCATGGTGCT	qRT-PCR
LRNA-QF	CCAGATGCTTGGCGTCCT	qRT-PCR
LRNA-QR	ACGCTTAACAACAAAACC	qRT-PCR
LRNA RT	CCAGATGCTTGGCGTCCTCTTTGCAACTGACGATGT	cDNA synthesis

### Analysis of Viral Protein Synthesis by Western Blotting

Monolayer of NA cells cultured in six-well plates were infected with rHep-Flury and variant viruses at a MOI of 3 and incubated at 34°C for RNAs analysis. At 12 post-infection, cells were washed in PBS once and lysed with RIPA buffer (containing 1× protease inhibitor cocktail) (Beyotime Biotech, China) on ice for 30 min. The suspension was then transferred to a microcentrifuge tube and spun for 20 min at 15,000 × *g* to remove all cell debris, before the suspension was quantified using a Pierce BCA Protein Assay Kit (Thermo scientific, USA). Proteins were separated by SDS-10% polyacrylamide gel electrophoresis (SDS-10% PAGE) and then transferred onto a polyvinylidene difluoride (PVDF) membrane (Millipore, USA). Blots were blocked in 5% dry milk powder in PBS for 1h. After blocking, blots were washed twice with a 0.1% PBS-Tween 20 solution and incubated overnight at 4°C with monoclonal mouse anti-RV N (Tongdian Biotech, Hangzhou, China) (diluted 1:1000), P (prepared in our lab) (diluted 1:500), M (prepared in our lab) (diluted 1:100) or G (prepared in our lab) (diluted 1:500), respectively. The β-actin (1:1000, Beyotime Biotech, China) was as the reference protein. Subsequently, blots were then washed four times with 0.1% PBS-Tween 20. Secondary goat anti-mouse horseradish peroxidase-conjugated antibodies (Bioworld Technology, USA) (diluted 1:50,000) were added, and blots were incubated for 2 h at 37°C. Blots were washed four times with 0.1% PBS-Tween 20 and once with PBS. Chemiluminescence analysis using BeyoECL plus (Beyotime Biotech, China) was performed as instructed by the vendor.

### Statistical Analysis

All results were expressed as the mean ± standard deviation (SD) and all statistical analyses were performed with one-way or two-way analysis of variance (ANOVA). Asterisks denote statistical differences (^∗^*P* < 0.05; ^∗∗^*P* < 0.01; ^∗∗∗^*P* < 0.001; ^∗∗∗∗^*P* < 0.0001) between different groups. A *P*-value of less than 0.05 was considered statistically significant. The statistical significance of survival rates was determined by the log-rank test and Kaplan–Meier survival analysis.

## Results

### Recovery of Rearranged Viruses

**FIGURE 1:**
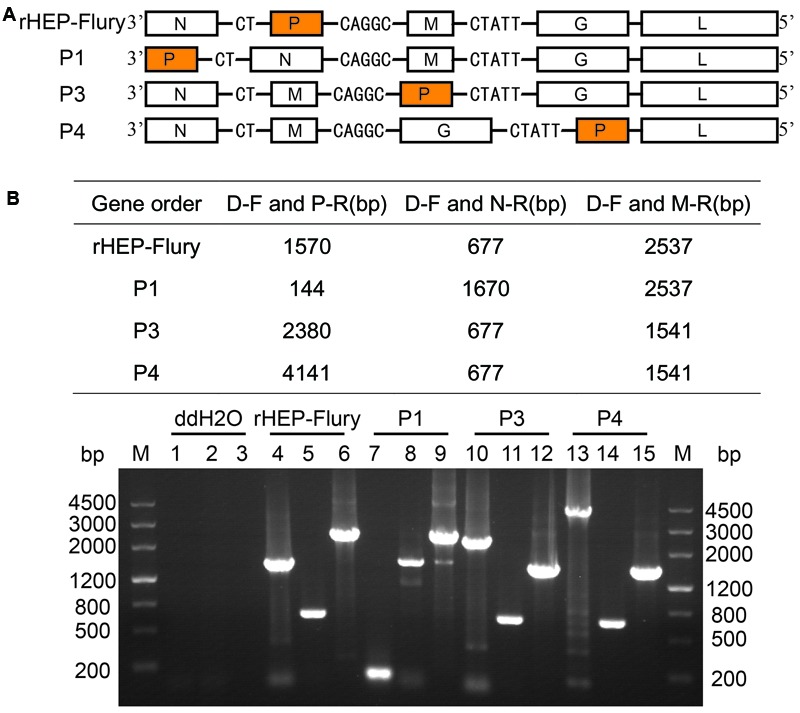
**Determination of gene order in recovered viral genomes. (A)** Schematic representations of the gene order of rHep-Flury and the rearranged variant viruses. **(B)** Genomic RNA was isolated from recovered viruses and analyzed by reverse transcription and PCR with different primer pair. Fragments were analyzed by electrophoresis on a 1% agarose gel in the presence of ethidium bromide. Lane M = marker DNA fragments with sizes as indicated. Lanes 1, 4, 7, 10, and 13: PCR products with primers D–F and P–R; Lanes 2, 5, 8, 11, and 14: PCR products with primers D–F and N–R; Lanes 3, 6, 9, 12, and 15: PCR products with primers D–F and M–R.

We rearranged the P gene of HEP-Flury by manipulating an infectious cDNA clone to translocate it from its normal position 2 to 1, 3, or 4 in gene order and rescued them successfully (**Figure 1A**). All other aspects of the viral nucleotide sequences remained unaltered.

The gene orders for each of the recovered viruses were verified after eight passages by RT-PCR carried out using three pairs of primers. The observed sizes of the amplified products were exactly as predicted (**Figure 1B**) and further direct sequencing demonstrated that they were specific bands. These data indicated that gene orders of the recovered viruses were as originally constructed and remained so after eight passages in cell culture.

### Lethality in Suckling Mice

**FIGURE 2:**
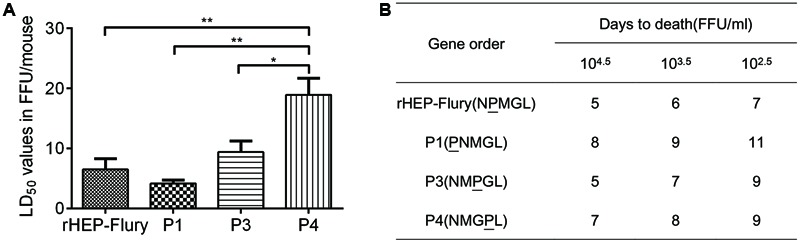
**Lethality of rHEP-Flury or rearranged viruses for suckling mice. (A)** The LD_50_ was calculated from mortality among groups of 12 mice inoculated IC with five serial, 10-fold dilutions of virus. The LD_50_ was analyzed for statistical significance by one-way ANOVA. Asterisks denote statistical differences (^∗^*P* < 0.05; ^∗∗^*P* < 0.01; ^∗∗∗^*P* < 0.001; ^∗∗∗∗^*P* < 0.0001) between different groups. **(B)** Onset of death after intracerebral inoculation of three serials.

Pathogenicity of recombinant RABVs were assessed in suckling mice as HEP-Flury was fatal for suckling mice but not for the adult mice following IC inoculation (Takayama-Ito et al., 2006). Suckling mice aged 1-day to 3-days served as a sensitive model to compare the relative lethality of rHEP-Flury and its mutants. By IC inoculation, the LD_50_ dose of P4 was significant higher than others. Moreover, the LD_50_ dose of rHEP-Flury increased by 1.7-fold, P3 increased by 2.3-fold, and P4 increased by 4.6-fold compared to P1 (**Figure 2A**). This declared that the LD_50_ dose was going to be higher as the P gene was moved successively away from promoter proximal position to successive positions down the viral genome.

The time to onset of death at doses of 10^4.5^ FFU/ml to 10^2.5^ FFU/ml per mouse are shown in **Figure 2B**. The rHEP-Flury-infected mice first appeared death at day 5 post-inoculation. Recombinant P3 elicited reproducibly pathogenesis as fast as rHEP-Flury-infected animals, whereas the onset of death from infection with P1 and P4 occurred later as the rule was more clearly with decreasing dose.

### Ability of Rearranged Viruses to Protect Against Wild-Type Challenge

**FIGURE 3:**
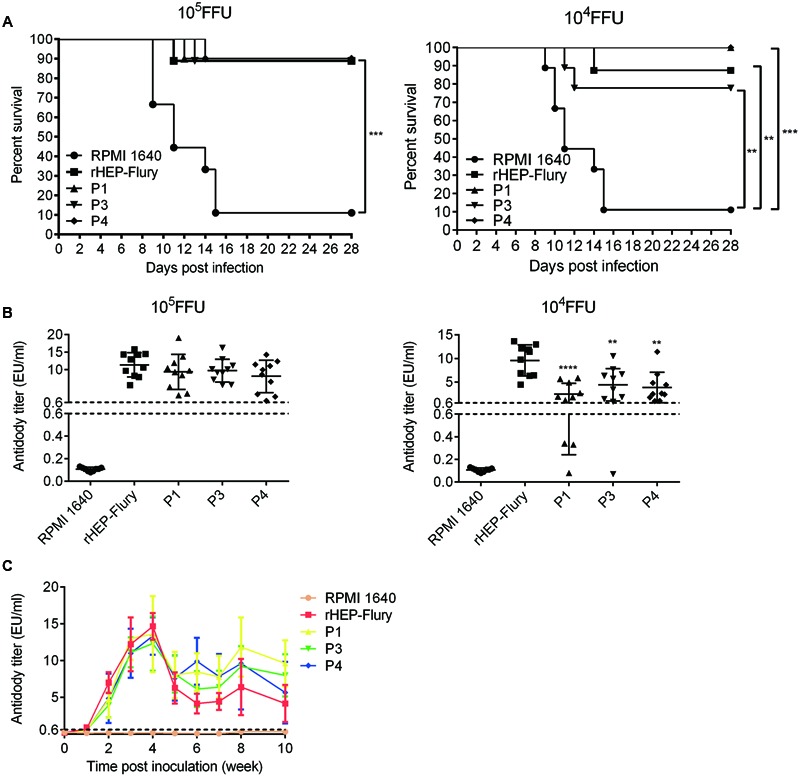
**Comparison of antibody production and ability to protect against lethal challenge. (A)** Survivorship of mice after immunized with rHEP-Flury, P1, P3, P4, or medium by IM route. The survival rates were analyzed for statistical significance by Kaplan–Meier plots (*n* = 10 in each groups; by log-rank test). **(B)** The presence of anti-RABV antibody in serum at 21 days post-inoculation prior to challenge. The serum antibody titer of P1, P3, or P4 was compared to rHEP-Flury using ordinary one-way ANOVA. Asterisks denote statistical differences (^∗^*P* < 0.05; ^∗∗^*P* < 0.01; ^∗∗∗^*P* < 0.001; ^∗∗∗∗^*P* < 0.0001) between different groups. **(C)** Antibody kinetics of viruses with P gene translocations. Groups of five mice were inoculated with 10^5^ FFU of each virus via IM injection. Serum samples were pooled and heated to 57°C for 30 min to inactivate complement. The serum antibody titers were monitored using a Serelisa^®^ Rabies Ab Mono Indirect kit.

To test whether the P gene translocation affected the ability to elicit a protective immune response, mice were immunized by IM inoculation with 10^5^ or 10^4^FFU of either rHEP-Flury or the variant viruses. The surviving animals were challenged 21 days later by IC inoculation with 50LD_50_ of CVS-24. The protections of all viruses were significantly higher than control groups and there was no significant difference between them (**Figure 3A**). We guessed that the P gene rearranged viruses all contained the wild type complement of genes which could induce a protective host response (Wertz et al., 1998).

At a dose of 10^5^FFU, P gene rearranged viruses showed the same protections as rHEP-Flury (**Figure 3A**). Consistent with this, there was no significant difference in the serum antibody titer between variant viruses and rHEP-Flury in the immunized animals prior to challenge on day 21 (**Figure 3B**). At a dose of 10^4^FFU, the antibody titers of viruses were consistent with the tendency of vital titer at a MOI of 0.01. This suggested that the antibody production was affected by the viral replication. Moreover, the survival rates of P1 and P4 were 100% little better than P3 (77.78%) or rHEP-Flury (87.5%), though their antibody titers were reverse at the dose of 10^4^FFU (**Figures 3A,B**). These data revealed that P gene rearranged viruses elicited a protective response that remained undiminished compared to that of the parent virus.

### Duration of Antibody Levels in Mice

Duration of antibody levels is essential for rabies vaccine when a single immunization is conducted. The antibody levels of the serum revealed similar antibody kinetics for all viruses. They reached a maximum at week 4 and then decreased and remained a level more than 0.6EU/ml during 10 weeks post-infection (**Figure 3C**). However, P gene rearranged RABVs showed higher antibody levels than rHEP-Flury 5 weeks post immunization. We speculated that the antibody titers fell evidently at week 5 as the viruses clearance by antibody occurred mainly during this time. And the antibody titer 5 weeks post immunization revealed the final balance between viral replication and antibody development *in vivo*.

### Effects of P Gene Rearrangement on Viral Replication and Spread

**FIGURE 4:**
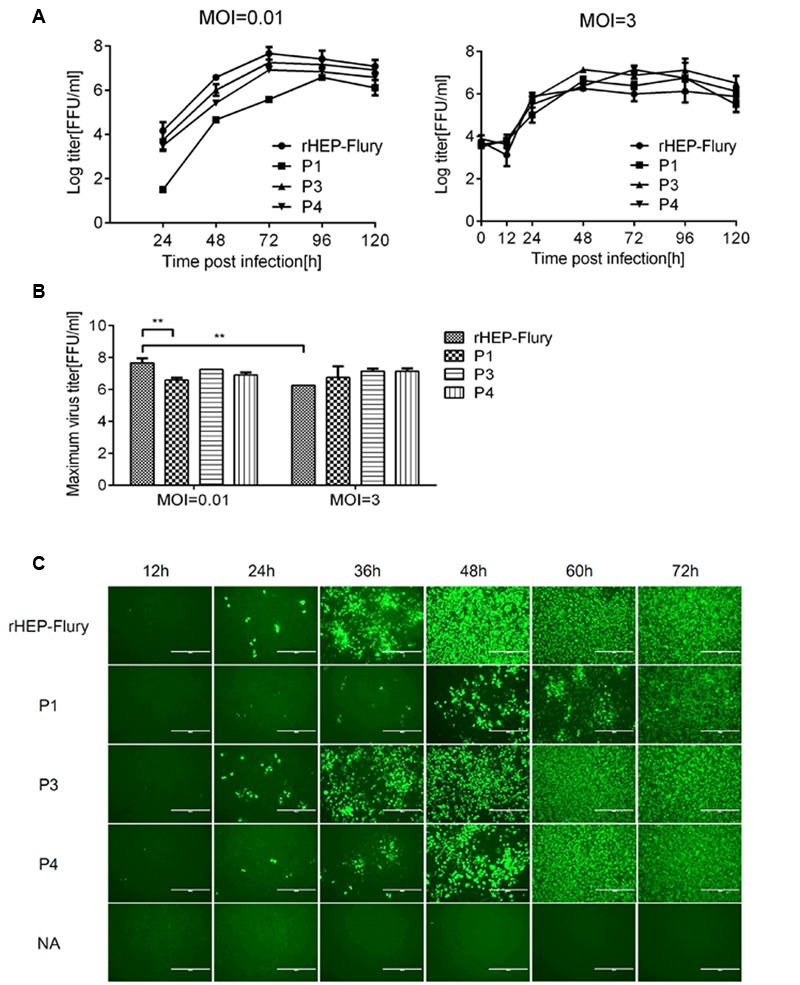
**Growth kinetics and spread of RABV strains in NA cells. (A)** Multi-step and one-step growth curves of the rHEP-Flury and recombinant strains were shown. NA cells were infected at a MOI of 3 (one-step) or 0.01 (multi-step) and incubated at 34°C for 120 h. Titers of virus harvested at the indicated intervals were assayed in triplicate, and the results were averaged. **(B)** The maximal titers of RABV strains in NA cells at a MOI of 0.01 or 3. The maximal titers were analyzed for statistical significance by two-way ANOVA. Asterisks denote statistical differences (^∗^*P* < 0.05; ^∗∗^*P* < 0.01; ^∗∗∗^*P* < 0.001; ^∗∗∗∗^*P* < 0.0001). **(C)** Viral spread in NA cells. NA cells were infected with every virus at a MOI of 0.005 for 72 h at 34°C, stained with FITC-conjugated monoclonal antibodies against RABV nucleoprotein, and examined under a fluorescence microscope. For each virus, three representative images taken from different areas of the same well and one of them were shown.

Slower replication and faster spread could enhance the RABV pathogenicity (Faber et al., 2005; Davis et al., 2015). We investigated viral replication and spread caused by P gene rearrangement in NA cells to further illustrate changes of lethality and immune response. Analysis of progeny virus production in cell culture revealed a decreasing ability to replicate at a MOI of 0.01 due to P gene translocation from its wild-type position 2 to 3 or 4 as predicted (**Figure 4A**). P1, which N gene was in position 2, had the worst ability to replicate and its maximum titer was significant lower than rHEP-Flury. At a MOI of 3, the P gene position affected the speed of growth at early stage. There was no significant difference in maximum titers between them (**Figure 4B**). However, the maximum titer of rHEP-Flury at a MOI of 0.01 was higher than that at a MOI of 3. Viral spread in NA cells also varied as grew at a MOI of 0.01(**Figure 4C**).

### Cell Apoptosis

**FIGURE 5:**
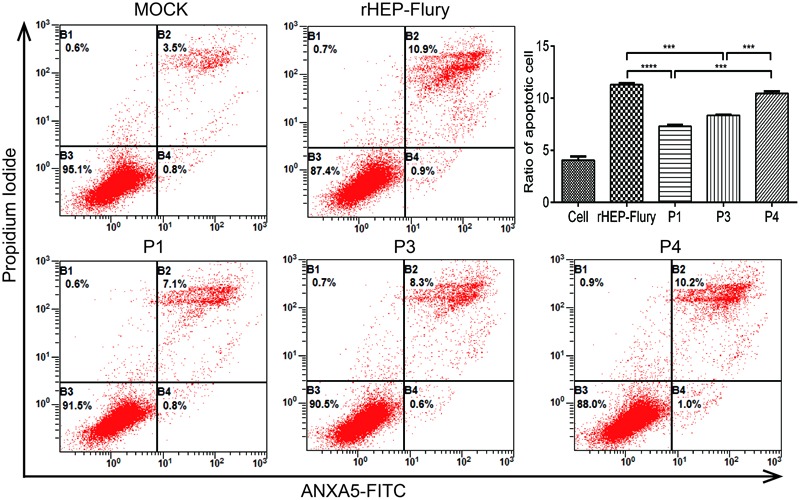
**Cell apoptosis induced by rHEP-Flury and rearranged viruses.** NA cells were infected with rHEP-Flury and rearranged viruses at a MOI of 3. Cell apoptosis was quantified by using Annexin V-FITC apoptosis kit. Cells in early apoptosis and dead cells were represented as the percentage of ANXA5-FITC and Ptdlns cells of total cells. Mean ± SD of three independent experiments. One-way ANOVA: ^∗∗∗^*P* < 0.001; ^∗∗∗∗^*P* < 0.0001.

Rabies virus HEP-Flury could induce NA cell apoptosis though RABV dose not induce a typical CPE in NA cells. Cell apoptosis is a particular factor attenuating the pathogenic potential of RABV (Préhaud et al., 2003; Thoulouze et al., 2003; Kassis et al., 2004). Previous work has shown that, at a MOI of 0.01, rHEP-Flury does not cause toxicity in NA cells, and only induced 2.9% NA cell apoptosis at 48 h post-infection (Yang et al., 2015b; Peng et al., 2016). In this study, at a MOI of 3, the percentage of early stage apoptotic cells as well as late stage apoptotic or even necrotic cells was about 11.8%. And P4 induced similar NA cell apoptosis as rHEP-Flury. They were significant more than that induced by P1 or P3 (**Figure 5**). We found that when G gene was in the same position, NA cell apoptosis was positive to the viral replication. The P4 induced more cell apoptosis as the G gene was moved one position closer to the promoter. This indicated that both the viral replication and G gene position were related to cell apoptosis induced by HEP-Flury.

### Effects of P Gene Rearrangement on Expression of RNAs and Proteins

Rabies virus is a neurotropic virus (Ugolini, 2011). We analyzed the synthesis of viral RNAs and proteins in infected NA cells to ascertain how P gene translocation affected viral gene expression, thus influencing the phenotype of RABV. Twelve hours post-infection, we stained the NA cells with FITC Anti-Rabies Monoclonal Globulin to confirm the infection of every cell.

**FIGURE 6:**
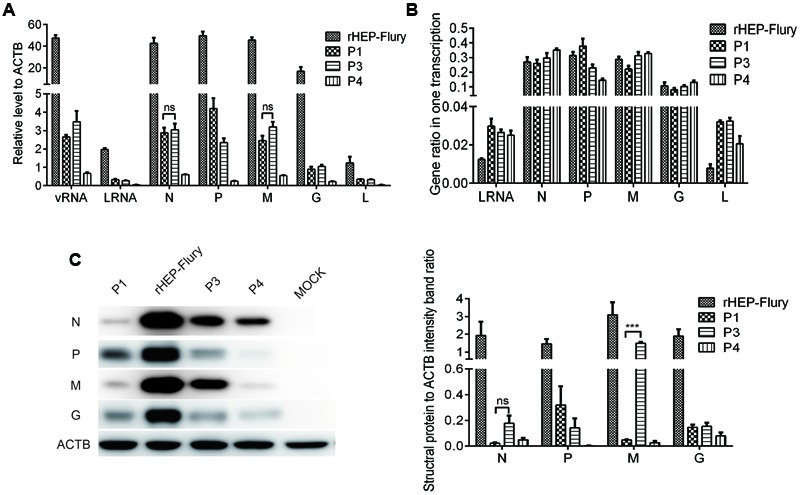
**Gene expression in NA cells.** NA cells were infected with rHEP-Flury, P1, P3 or P4 at a MOI of 3 for 12 h at 34°C. **(A)** qRT-PCR analyses of RNAs level. The relative amount of individual viral RNAs was normalized in relation to the housekeeping gene β-actin. Data are mean ± SD, *n* = 3. **(B)** Ratios of leader RNA, N mRNA, P mRNA, M mRNA, G mRNA, or L mRNA in one complete transcription. Individual RNA ratio were calculated relative to all structural genes plus leader RNA: leader RNA+N+P+M+G+L. Data are mean ± SD, *n* = 3. **(C)** Viral structural protein were quantified by Western blotting with monoclonal antibody against RABV N, P, M, G, and a monoclonal antibody against actin. Densitometry of the western blotting was analyzed with the Image-Pro Plus 6.0 software.

RNA and protein profiles of cells infected with the rHEP-Flury and variant viruses showed that both the RNAs and protein levels of rHep-flury were the most at 12 h post-infection (**Figure 6**). That was rHep-flury with the wild-type gene order was always the most fit for growth and gene expression first had to meet the requirement of sufficient virus replication (Finke and Conzelmann, 2005). P mRNA substantially decreased as its gene was moved successively away from the promoter in viruses P1, P3, and P4. The transcription levels of N, M, G and L mRNA were reduced as the vRNA reduced presumably as a secondary effect because of the decrease in replication (**Figure 6A**). Correlation analysis revealed they had significant correlation (correlation coefficients >0.9, *P* < 0.001). RABV only encoded five subgenomic mRNAs that were translated to yield five proteins, all of which were components of the mature virion (Okumura and Harty, 2011b). And at this time, the viruses did not budding from the cells (**Figure 4A**), so they had tightly relationship. However, at 24 and 48 h post-infection, when the viruses budding from the cells which we could found in one-step curve, vRNA levels were on behalf of the balance between synthesis and budding while the level of N, P, M, G, and L mRNA only showed the synthesis, so there was no significant correlation between them (**Supplementary Figure S1**).

Then we analyzed the gene ratio in one complete transcription for each virus, i.e., the ratios of viral structural RNAs were calculated relative to all structural genes plus leader RNA: leader RNA+N mRNA+ P mRNA+ M mRNA+ G mRNA+ L mRNA in every virus. The data showed that the ratio of P mRNA decreased as its gene was moved successively away from the promoter in viruses P1, rHEP-Flury (P2), P3, and P4 (**Figure 6B**). Consistent with this decrease, a decrease in the ratio of N mRNA was also observed with virus P1 in which the N gene was moved one position farther to the promoter; an increase in the ratio of M mRNA was observed with virus P3 or P4 in which the M gene was moved closer to the promoter; an increase in the ratio of G mRNA was observed with virus P4 in which the G gene was moved closer to the promoter (**Figure 6B**). These were as predicted by the model of progressive transcriptional attenuation though previous work has shown that N mRNA and P mRNA of HEP-Flury were qualitatively similar in infected BHK cells (Morimoto et al., 2011). We speculated that gene transcription was also associated with the gene. Moreover, the ratios of leader RNA and L mRNA increased as P gene translocated though their positions in gene order without change. This was an interesting and important observation.

The amounts of viral proteins were qualitatively similar with their mRNAs as the translation efficiency was mainly regulated by the level of transcription (**Figure 6C**). However, the N and M translation efficiency of P1 were lower than others. There might be some other factors inhibiting the translation when the N gene was translocated from position 1 to position 2, following the P gene translocation. Moreover, G protein levels in NA cells infected by P gene rearranged viruses were more abundant than that infected by rHEP-Flury (**Supplementary Figure S1**). This might induced more effective virus neutralizing antibody (VNA), thus increased the protection of mice.

## Discussion

The results presented above revealed that the RABV P gene can be rearranged from its wild type position 2 to 1, 3, and 4 in the genome, leading to successful recovery of infectious viruses. Subsequently, the data showed that gene orders of the recovered viruses correspond to the cDNA clones from which they were recovered. There was no evidence of reappearance of the wild-type genetic order among the variants. As a consequence, it further proved that gene rearrangement should be viable and irreversible (Pringle, 1982). However, the *in vivo*- and *in vitro*-characteristics of rearranged RABVs were different though they could be rescued successfully.

P protein of RABV could interrupt the IFN transcription, consequently increased viral pathogenicity (Kuang et al., 2009; Niu et al., 2013). As our results shown, the viral lethality for suckling mice was in accord with the ratio of P mRNA in one complete transcription which decreased as its gene was moved successively away from promoter proximal position to successive positions down the viral genome, though the absolute value of P mRNA or protein was not consistent with this. Meanwhile, we found that the RABV P4 which G mRNA ratio increased, its pathogenicity was significant weaker than others. As HEP-Flury G protein could induce cell apoptosis which was contribute to attenuate the pathogenicity of RABV (Yang et al., 2015b; Peng et al., 2016). We speculated that the pathogenicity of RABV was correlated with the ratio of viral gene. Moreover, fast spread was conceived to viral escaping from antiviral host cell response, thus enhancing the RABV pathogenicity (Faber et al., 2005; Takayama-Ito et al., 2006). Here spread speed mainly affected the onset time of death in suckling mice. We suggested that the viruses have kept away from the majority of the host immune response when inoculated to suckling mice by IC.

Serum antibody levels at 21 days post-inoculation induced by P gene rearranged viruses were lower than rHEP-Flury as their replication efficiency reduced. However, the protections of P1 and P4 were little better than rHEP-Flury and P3 while their antibody titers were reverse. Duration of antibody levels in mice showed that P gene rearranged RABVs had higher antibody levels than rHEP-Flury 5 weeks post-immunization which might be the effective antibody used to remove the RABV CVS-24. Meanwhile, we found that the antibody levels were consistent with the G protein levels in infected NA cells which could induce VNA (Faber et al., 2002; Li et al., 2006). Moreover, the leader RNA ratio of P gene rearranged viruses increased which was contribute to activate dendritic cells (Kammouni et al., 2015), thus enhancing the protection of mice.

To further illustrate impacts on replication, spread and cell apoptosis by P gene rearrangement, we evaluated them in NA cells. Firstly, we found that vRNA replication varied as their N mRNA levels, though P protein was a noncatalytic cofactor for the polymerase L and conferring the specificity of genomic RNA encapsidation by N (Emerson and Yu, 1975; Liu et al., 2004). This declared N protein played the most important role in viral replication (Albertini et al., 2011; Choi et al., 2015). Though N genes of rHEP-Flury, P3 and P4 were all in position 1, the N gene expression decreased as the P gene was translocated from position 2 to 3 or 4. We suggested optimal N: P: L ratio also had to achieve optimal RNA replication in RABV which has been demonstrated in VSV (Pattnaik and Wertz, 1990). And the P gene rearranged viruses regulated the N gene expression in order to reach the optimal N: P: L to facilitate the viral replication. This might be caused by regulating the binding between N protein and nascent leader RNA (Albertini et al., 2011). The structural M protein of RABV which was an essential factor for virus budding was not only a regulatory protein adjusting the balance of RNP replication and mRNA synthesis but also regulating the viral replication start. (Finke and Conzelmann, 2003; Finke et al., 2003; Davis et al., 2015). Consistent with this work, we found that the replication of rHEP-Flury and P3 were faster than P1 or P4 as their M protein levels were also higher at 12 h post-infection. As for P1, the reduction in viral replication was not only due to the vRNA synthesis but also the inhibition of virion release.

G protein was a key element for viral spread in CNS and a G gene deletion-mutant RABV cannot spread beyond initially infected cells (Takayama-Ito et al., 2006; Wickersham et al., 2007; Beier et al., 2013). But as for HEP-Flury, G protein could induce the apoptosis of NA cells (Liu et al., 2014; Yang et al., 2015b), consequently limiting the viral spread in CNS (Lay et al., 2003; Sarmento et al., 2005, 2006). Here the results indicated that the viral replication mainly affected efficiency of cell-to-cell spread but not apoptosis-inducing ability.

Analysis of viral RNAs and proteins showed that the ratio of N, P, M, or G mRNA in one transcription was tightly related with gene position though their absolute values were mainly affected by viral replication. We speculated that gene position mainly regulated the gene ratio in one transcription. Moreover, we found that the ratios of leader RNA and L mRNA increased when P gene was translocated. Leader RNA can bind La protein which may inhibit cellular RNA synthesis (Kurilla et al., 1984), thus inhibited the viral replication. And L protein of an attenuated vaccine strain SAD B19 can bind to a dynein light chain 1 (DLC1) acted as a transcription enhancer (Bauer et al., 2015). Both of them decreased the viral replication and stimulated the viral transcription.

In summary, these results revealed that P gene rearrangement of RABV was viable. Viral replication was affected by the absolute value of N protein which was regulated by P protein. Viral lethality in suckling mice was consistent with the ratio of P mRNA in one complete transcription. The protection of mice induced by viruses was related to the antibody titer 5 weeks post-inoculation which might be regulated by G protein. However, the ability to induce cell apoptosis and viral spread were not only related to the viral replication but also to the ratio of related gene which was consistent with the gene position. Subsequently, based on these, we could construct the optimizing RABV as a vaccine candidate and RABV was found lack the mechanism for homologous recombination, this should be an irreversible and stably approach.

## Author Contributions

MM performed the research and wrote the article; TL, QT, YW, and JL performed the technique of molecular biology; JZ and QZ did the animal experiments; JP provided analysis tools; YL contributed reagents/materials, and XG designed the research and assisted correction of the article.

## Conflict of Interest Statement

The authors declare that the research was conducted in the absence of any commercial or financial relationships that could be construed as a potential conflict of interest.

## Supplementary Material

FIGURE S1 **Gene expression in NA cells.** NA cells were infected with rHEP-Flury, P1, P3 or P4 at a MOI of 3 for 24 h **(A)** and 48 h **(B)** at 34°C. Then RNAs level and viral structural protein were analyzed by qRT-PCR and western blotting respectively. The relative amount of individual viral RNAs was normalized in relation to the housekeeping gene β-actin. Individual RNA ratio were calculated relative to all structural genes plus leader RNA: leader RNA+N+P+M+G+L. Viral structural protein were quantified by Western blotting with monoclonal antibody against RABV N, P, M, G and a monoclonal antibody against actin. Densitometry of the western blotting was analyzed with the Image-Pro Plus 6.0 software. Data are mean ± SD. *n* = 3.
